# Disinfectant Treatment of Small-Pox

**Published:** 1872-03

**Authors:** 


### Disinfectant Treatment of Small-Pox.

Dr. Stevens writes to the *British Medical Journal:*

Having lately had charge of two small-pox hospitals in Plymouth, I have adopted the disinfectant treatment of Dr. Sansom, of London, viz., the administration internally of bi-sulphate of soda every four hours, and the application of olive-oil and carbolic acid to the pustules externally. By these means he contends, that "the patients are themselves disinfected, and rendered inocuous to the community at large." I must say that this plan has been attended by more favorable results than any other with which I had previously been acquainted, and can most strongly recommend it to my professional brethren. I have, however, to make certain that no infection should be conveyed to the public after leaving the hospital, caused each patient to be put for a quarter of an hour on three successive days into a bath containing a pint of chloralum. This has been done immediately convalescence has been established, and the patients have been discharged from the institution forthwith. By this proceeding much expense has been saved, and the beds made available for other sufferers in quick rotation. I have also employed chloralum in solution as an external remedy before the application of olive-oil and carbolic-acid. This has been the means of cooling the inflamed surface and of allaying the itching. *Medical and Surgical Reporter.*

